# What makes a lonely child: environmental, health, and multimodal neuroimaging correlates of prospective loneliness in the ABCD study

**DOI:** 10.1111/jcpp.70178

**Published:** 2026-05-25

**Authors:** Ting Yat Wong, Ting Sam Wong, Wai Kai Hou, Angel Nga Man Leung, Jie Xiao, Kenneth S. L. Yuen, Simon S. Y. Lui, Anqi Qiu, Tyler M. Moore, Ruben C. Gur

**Affiliations:** ^1^ Department of Psychology The Education University of Hong Kong Hong Kong Special Administrative Region China; ^2^ Centre for Psychosocial Health The Education University of Hong Kong Hong Kong Special Administrative Region China; ^3^ AI, Brain and Child Research Centre (ABC‐RC), Academy of Educational Development and Innovation The Education University of Hong Kong Hong Kong Special Administrative Region China; ^4^ SWU‐EdUHK Joint Lab for Research on Brain, Education, and Intelligence (BEI Lab) Hong Kong Special Administrative Region China; ^5^ Independent Data Analyst Nagoya City Japan; ^6^ Analytics\Assessment Research Centre The Education University of Hong Kong Hong Kong Special Administrative Region China; ^7^ Focus Program Translational Neuroscience, Neuroimaging Center Johannes Gutenberg University Medical Center Mainz Germany; ^8^ Leibniz Institute for Resilience Research Mainz Germany; ^9^ Department of Psychiatry, School of Clinical Medicine The University of Hong Kong Hong Kong Special Administrative Region China; ^10^ Department of Biomedical Engineering National University of Singapore Singapore Singapore; ^11^ Department of Health Technology and Informatics Hong Kong Polytechnic University Hong Kong Special Administrative Region China; ^12^ Department of Biomedical Engineering Johns Hopkins University Baltimore MD USA; ^13^ Department of Psychiatry, Brain Behavior Laboratory Perelman School of Medicine, University of Pennsylvania Philadelphia PA USA; ^14^ Lifespan Brain Institute (LiBI) Children's Hospital of Philadelphia and Penn Medicine Philadelphia PA USA

**Keywords:** Prospective loneliness, late childhood, environmental and health factors, neuroimaging correlates

## Abstract

**Background:**

Loneliness in childhood is a growing public health concern, yet early multilevel candidate risk and protective factors remain insufficiently mapped. Systematic investigation is essential to guide prevention and intervention during sensitive developmental periods. This study identifies environmental, health, and neurobiological factors associated with prospective loneliness in children.

**Methods:**

A population‐based longitudinal cohort study used data from children aged 9–10 years and their caregivers enrolled in the Adolescent Brain Cognitive Development (ABCD) Study between 2016 and 2022. A total of 9,602 children with complete baseline and follow‐up loneliness and demographic data were included after exclusions for exposure completeness and quality assurance. Baseline measures included 347 environmental exposures, 61 health indicators, and 558 MRI features capturing gray matter volume, white matter microstructure, and resting‐state functional connectivity. The primary outcome was prospective loneliness reported from ages 10–14. Linear mixed‐effects models assessed associations with environmental and health variables. Linear discriminant analysis was applied to neuroimaging features to distinguish children with and without prospective loneliness.

**Results:**

Among 9,602 children (mean [SD] age, 119.01 [7.52] months; 48% girls; 55% White, 14% Black, 31% other races/ethnicities), 12% (*n* = 1,158) reported loneliness at baseline, and 71.6% (*n* = 829) of those re‐experienced loneliness over 3 years. Prospective loneliness was significantly associated with 40 environmental variables (|*d*| = 0.069–0.388), most strongly parental psychopathology, developmental history, and family income. Twenty‐six health indicators were also associated (|*d*| = 0.061–0.390), with general mental health showing the largest effect. Neuroimaging features associated with prospective loneliness converged in brain systems involved in socioemotional processing.

**Conclusions:**

Prospective loneliness was associated with modifiable environmental and health factors, as well as neurobiological differences. Early identification and targeted interventions that support socioemotional development, particularly within family, neighborhood, and school contexts, may help mitigate loneliness and its long‐term impact.

## Introduction

Social health, alongside physical and mental health, is a foundational pillar of human well‐being and critical for children to thrive (Lau, Priebe, & Morgan, 2025). Loneliness, a key indicator of social health, is characterized by a subjective experience marked by a perceived unmet need of desired social relationships (Hawkley, 2022; Heinrich & Gullone, 2006). It has been empirically linked through numerous meta‐analyses to a spectrum of adverse health outcomes, including impaired cognitive development (Samtani et al., 2022), poorer mental health (Park et al., 2020; Solmi et al., 2020; Wang, Mann, Lloyd‐Evans, Ma, & Johnson, 2018), increased suicidality (McClelland, Evans, Nowland, Ferguson, & O'Connor, 2020), more sleep problems (Hom, Chu, Rogers, & Joiner, 2020), and a heightened risk of mortality (Wang et al., 2023). Loneliness affects individuals of all ages globally and is especially common among adolescents (12–17 years), with prevalence rates ranging from 9.2% in South‐East Asia to 14.4% in the Eastern Mediterranean region (Surkalim et al., 2022). In children as young as 8 years old, the prevalence of loneliness could be as high as 20% (Lempinen, Junttila, & Sourander, 2018). A global study involving over one million adolescents found that rates of loneliness nearly doubled between 2012 and 2018 (Twenge et al., 2021). These concerns were further amplified during the COVID‐19 pandemic, when social distancing policies, school closures, and reduced peer contact led to substantial increases in loneliness among children and adolescents (Farrell, Vitoroulis, Eriksson, & Vaillancourt, 2023). Despite these concerning trends, there remains a limited body of research examining factors associated with prospective loneliness in children (Buecker et al., 2024). Systematically identifying risk and protective factors is a critical step toward informing timely, evidence‐based prevention and intervention strategies.

The environment plays a pivotal role in determining loneliness (Astell‐Burt et al., 2022; Bower et al., 2023) over and beyond individual psychosocial and demographic factors (Barjaková, Garnero, & d'Hombres, 2023; Schnepf, Boldrini, & Blaskó, 2023). Some argue that loneliness stems from insufficient prioritization of individual health and social needs within urban planning and societal systems (Feng & Astell‐Burt, 2022). Numerous studies have also underscored the multilevel determinants of loneliness, emphasizing the necessity for interventions that go beyond individual considerations (Barjaková et al., 2023; Marquez et al., 2023). However, previous research could be constrained by heterogeneous definitions of environmental concepts, such as self‐report methods, different geographical scales, and temporal considerations, limiting the transferability of the findings to adolescents across regions (Bower et al., 2023). To improve generalizability, studies using consistent environmental metrics within large, population‐based cohorts are needed.

The transition from late childhood to adolescence is a crucial developmental period characterized by significant biological, psychological, and social changes (Casey, Getz, & Galvan, 2008). This phase is pivotal for the maturation of higher‐order functioning, specifically for executive functions and social cognition (Blakemore, 2012; Fuhrmann, Knoll, & Blakemore, 2015; Larsen & Luna, 2018; Orben, Tomova, & Blakemore, 2020; Paus, 2005). The continuous reduction of gray matter volume and simultaneous increase of white matter volume during this period (Bethlehem et al., 2022) demonstrates an ongoing reconfiguration of the brain to further refine cognition. Accordingly, the brain network modules become more segregated to support the development of executive function during adolescence (Baum et al., 2017). Any deviations from typical brain development could be associated with poorer psychological outcomes (Bethlehem et al., 2022; Wong et al., 2023). Loneliness was consistently associated with altered brain structures or activities within the prefrontal cortex, insula, amygdala, hippocampus, and posterior superior temporal cortex (Cacioppo, Capitanio, & Cacioppo, 2014; Lam et al., 2021). These regions form a distributed network implicated in key socioemotional processes (Blakemore, 2008). However, neuroimaging studies of loneliness have generally relied on modest sample sizes and predominantly adult cohorts, limiting developmental inference and generalizability to late childhood and early adolescence. In contrast, large‐scale, well‐characterized population‐based cohorts offer a stronger framework for examining robust and generalizable brain–behavior associations across development (Kuang et al., 2023; Liu et al., 2025; Wu et al., 2025). Accordingly, large‐sample studies are still needed to clarify the neurobiological correlates of loneliness in late childhood and early adolescence.

Despite growing attention, research on loneliness in late childhood remains limited, particularly regarding prospective risk and protective factors with longitudinal designs (Binte Mohammad Adib & Sabharwal, 2023; Mann et al., 2017; Marquez et al., 2023). In this study, we aimed to systematically examine the environmental, health, and neurobiological correlates associated with the presence of prospective loneliness during late childhood and early adolescence in a large, well‐characterized cohort. Using a mass‐univariate association approach with multiple comparison adjustment, we investigated associations between prospective loneliness and a broad range of environmental and health variables in children. This approach helps avoid selective reporting and publication bias (Patel, Manrai, Corona, & Kohane, 2017). Additionally, we applied linear discriminant analysis to identify multimodal brain features that distinguish children with prospective loneliness. We hypothesized that prospective loneliness is associated with multilevel environmental and health factors and that alterations in the brain systems involved in socioemotional processing would also be associated with prospective loneliness.

## Methods

### Study design and data source

The ABCD baseline cohort initially comprised 11,868 children aged 9 to 10 years old between 2016 and 2018. Participants were recruited from 21 sites across the United States through school systems (Garavan et al., 2018). In the present study, participants were therefore drawn from a relatively narrow developmental window centered on late childhood and early adolescence. We incorporated information up to the 3‐year follow‐up to ensure comprehensive participant inclusion. Analyses were conducted from March 2024 to March 2025.

### Loneliness

We utilized a parent‐reported item (*cbcl_q12_p*) from the Child Behavior Checklist (CBCL) (Achenbach & Rescorla, 2001) to assess children's loneliness. This item evaluates whether the child has complained of loneliness in the past 6 months, with response options as follows: 0 = Not True; 1 = Somewhat/Sometimes True; 2 = Very True/Often True. Because our primary objective was to identify children at elevated risk of prospective loneliness rather than to model severity or persistence gradients, the CBCL item was dichotomized (0 vs. 1/2) to indicate the presence of loneliness. This approach was further justified by the rarity of the highest response category in this cohort (86.6% coded as 0, 12.2% as 1, and 1.3% as 2). In the present study, prospective loneliness was defined as any parent‐reported loneliness endorsed at the 1‐, 2‐, or 3‐year follow‐up assessments. Participants with missing data at any of these follow‐up time points were excluded to ensure a consistent definition of the prospective outcome.

### Environment

We included a comprehensive set of 347 environmental variables at baseline in the analyses. We further organized these variables into 13 categories, including Amenities and Services, Community Health Care, Developmental History, Family Values, Laws and Policies, Neighborhood Environment, Neighborhood Safety, Neighborhood Socioeconomic Status, Parental Psychopathology, Pollution, School Environment, Family Socioeconomic Status, and Urbanization. Details of each included variable are available in the Appendix S1.

### Health

A detailed description of 61 health indicators at baseline is available in the Appendix S1, while a brief overview is provided below.

#### Mental health

The CBCL (Achenbach & Rescorla, 2001) utilized T‐scores and evaluated mental health across eight syndrome dimensions (anxious/depressed, withdrawn/depressed, somatic, social, thought, attention, rule‐breaking, and aggressive), plus three summary variables (internalizing, externalizing, and total problems) to assess different levels of mental well‐being. Psychotic‐like experiences were assessed by the Prodromal Questionnaire – Brief Child Version (Karcher et al., 2018). Subsyndromal mania symptoms were measured by the Parent General Behavior Inventory (Youngstrom, Frazier, Demeter, Calabrese, & Findling, 2008).

#### Neurocognition

Neurocognitive performance was evaluated using the NIH Toolbox® Cognition Battery, including Dimensional Change Card Sort, Flanker Inhibitory Control and Attention, List Sorting Working Memory, Oral Reading Recognition, Pattern Comparison Processing Speed, Picture Sequence Memory, and Picture Vocabulary (Thompson et al., 2019; Weintraub et al., 2013). The outcomes of these seven tests were aggregated into composite scores, including a total cognitive score and separate scores for crystallized and fluid cognition. Additionally, the Little Man Test, assessing visuospatial processing through mental rotation, was incorporated into the task battery in the ABCD Study. The WISC‐V Matrix Reasoning measured fluid reasoning, part–whole spatial reasoning, visual intelligence, perceptual organization, sequencing, and attention to detail. The RAVLT was utilized to measure auditory learning, memory, and recognition.

#### Physical health

Anthropometric measurements of height and weight representing the average of up to three separate measures, and body mass index (BMI) (kg/m^2^) were calculated. Additionally, waist measurements were taken. The Sleep Disturbance Scale for Children (Bruni et al., 1996) assesses the frequency of disorders of initiating and maintaining sleep, sleep breathing disorders, disorders of arousal, sleep–wake transition disorders, disorders of excessive somnolence, and sleep hyperhidrosis in the past 6 months. The caregivers completed a medical history questionnaire about their children, adapted from the Missouri Assessment of Genetics Interview for Children Health Services Utilization Questionnaire (Todd, Joyner, Heath, Neuman, & Reich, 2003). It included information on various health conditions such as asthma, allergies, bronchitis, leukemia, cerebral palsy, diabetes, epilepsy, hearing loss, kidney disease, lead poisoning, muscular dystrophy, multiple sclerosis, vision problems, heart problems, sickle cell anemia, headache, previous operations, and other illnesses. A ‘total medical problems’ summary score was derived by summing the endorsed conditions for each participant. The self‐report Pubertal Developmental Scale (Petersen, Crockett, Richards, & Boxer, 1988) was completed by both children and their caregivers. An average pubertal development score was calculated based on the reports from both children and caregivers.

### Environment and health data preprocessing

All data preprocessing and statistical analyses were conducted using R version 4.3.1. Participants with more than 15% missing data were excluded. Binary variables with highly skewed distributions or minimal variation (i.e., less than 5% prevalence) were also excluded. Following data consolidation, such as aggregating individual items into composite scores (e.g., total trauma and total medical problems), a total of 294 unique environmental variables and 49 health measures were retained for analysis.

### Associations of environment and health with prospective loneliness

Mass‐univariate linear mixed‐effects models were used to assess associations between prospective loneliness and each environmental and health variable within an exposure‐wide association framework (Patel, Bhattacharya, & Butte, 2010), in which each independent variable was examined in a separate model. Linear mixed‐effects models are also commonly adopted as analytic strategies in prior large‐scale cohort studies (Fan et al., 2023; Yu et al., 2023). All models included baseline loneliness, age, sex, and race/ethnicity as covariates, along with random intercepts for site and family. Only core demographic covariates were included to maintain a consistent and parsimonious adjustment strategy, as many contextual and socioeconomic variables were evaluated as exposures. As such, the resulting estimates represent marginal associations and should not be interpreted as independent effects across correlated exposures. Mixed‐effects logistic regression models with a binomial error structure and logit link function were fitted, including random intercepts for study site and family to account for clustering. Log‐transformed odds ratios (ORs) were extracted and converted to Cohen's *d* to aid interpretation. To control for multiple comparisons, we applied the Bonferroni correction to provide stringent and transparent control of the family‐wise error rate in this large‐scale screening context, thereby prioritizing specificity and reducing false positives.

### Brain MRI data acquisition and preprocessing

Structural, diffusion, and resting‐state functional MRI data were collected, processed, analyzed, and quality assured according to previously established ABCD protocols (see Appendix S1 for more details) (Casey et al., 2018; Garavan et al., 2018; Hagler et al., 2019). In short, T1 images underwent gradient nonlinearity distortion correction through scanner‐specific transformations. Cortical reconstruction and volumetric segmentation were conducted by the ABCD Data Analysis, Informatics, and Resources Center (DAIRC) using FreeSurfer v7.1.1. The gray matter volume (GMV) was aligned to 34 cortical parcellations per hemisphere based on the Desikan‐Killiany atlas. Additionally, 19 subcortical segmentations were performed (Fischl et al., 2002). The DAIRC employed both automated and manual quality review processes before data sharing.

White matter tract labeling was conducted using AtlasTrack. Diffusion tensor imaging (DTI) was used to estimate microstructural properties, including fractional anisotropy (FA), axial diffusivity (AD), and radial diffusivity (RD). Standard linear estimation techniques, incorporating log‐transformed diffusion‐weighted signal intensities, were used to compute diffusion tensor parameters. DTI measures were averaged within AtlasTrack‐defined white matter tracts of interest.

Resting‐state fMRI data were acquired using an axial echo‐planar imaging sequence. Functional connectivity was assessed by computing pairwise correlations between ROIs within functionally defined parcellations (i.e., Gordon networks) and subcortical regions (i.e., cerebellum, thalamus, caudate, and putamen). Correlation values were Fisher Z‐transformed and analyzed within and between networks, as well as between networks and subcortical ROIs.

Only scans that had no clinically significant incidental findings (mrif_score = 1 or 2) and that passed ABCD quality control for T1‐weighted images (imgincl_t1w_include = 1) were included in the analysis. For diffusion and resting‐state fMRI data, inclusion also required passing modality‐specific quality control (imgincl_dmri_include = 1 for dMRI or imgincl_rsfmri_include = 1 for rsfMRI). For each neuroimaging feature, a linear mixed‐effects model was fitted to remove the influence of relevant covariates. Models included baseline loneliness, age, sex, race/ethnicity, and either intracranial volume (for GMV) or mean motion (for WMM and RSFC) as fixed effects, with random intercepts for MRI scanner ID and family ID. The resulting residuals were extracted, winsorized at ±3 standard deviations to limit the influence of outliers, and standardized (z‐scored) for downstream analyses.

### Identifying neural correlates of prospective loneliness with LDA

To identify and characterize neurobiological features associated with prospective loneliness, we conducted a multivariate analysis to differentiate individuals with and without prospective loneliness based on regional gray matter volume (GMV), white matter microstructure (WMM), and resting‐state functional connectivity (RSFC) separately. Given the high intercorrelation among MRI‐derived features, we used linear discriminant analysis (LDA) primarily as an inferential tool to characterize coherent multivariate distinctions between children with and without prospective loneliness, rather than to maximize individual‐level classification performance (Kopal et al., 2023; Zhou et al., 2025). This analytic approach follows prior large‐scale neuroimaging studies that applied LDA to derive an interpretable multivariate coefficient vector summarizing the simultaneous contribution of correlated features to a discriminant axis. Specifically, LDA provided a one‐dimensional coefficient vector for each imaging modality, reflecting the joint contribution of correlated features to group separation. Because all features were estimated jointly within a single multivariate model, this approach avoids the need for feature‐wise multiple‐comparison correction typical of mass‐univariate analyses. To quantify the contribution of individual features within each imaging modality, we implemented a bootstrapping procedure with 1,000 iterations. In each iteration, we performed random undersampling to balance group sizes and mitigate the effects of class imbalance. This procedure yielded bootstrap distributions of discriminant coefficients for each feature. A feature was deemed significant if the 95% bootstrap confidence interval (2.5th to 97.5th percentile) of its coefficient distribution excluded zero.

### Sensitivity analysis

Sensitivity analyses were conducted to evaluate the robustness of the primary findings. First, to assess temporal stability and determine whether the observed associations were driven by aggregation across multiple follow‐up waves, analyses were repeated using loneliness at the 1‐year follow‐up as the outcome. Second, to isolate incident cases and reduce potential confounding by pre‐existing loneliness, analyses were repeated in the subsample of participants without baseline loneliness. Third, to evaluate the potential influence of the COVID‐19 pandemic, analyses were restricted to participants whose first loneliness report occurred before the pandemic period. In addition, LDA‐derived patterns were compared with estimates from linear mixed‐effects models to evaluate convergence between multivariate and univariate analytic approaches. Further details are provided in the Appendix S1.

### Data and code availability

The data utilized in this study were sourced from the ABCD Study (https://abcdstudy.org), available through the NIMH Data Archive (NDA). The ABCD data repository is subject to growth and evolution over time. The current study utilized the data release 5.1 (https://doi.org/10.15154/z563‐zd24). All preprocessing and analysis scripts for the current study can be found in the corresponding GitHub repository: https://github.com/lonilab/abcd_prospective_loneliness.

## Results

After applying exclusion criteria, 9,602 participants were included (mean age = 119 ± 7.52 months; 48% girls). A flowchart detailing the participant inclusion process and number of participants in the main analysis is presented in Figure S1. Comparison of included and excluded participants suggested limited evidence of broad selection bias for age, sex, or baseline loneliness, although race/ethnicity differed between groups, indicating some demographic imbalance in the analytic sample (Table S1). Table 1 compares demographic characteristics between children who reported prospective loneliness and those who did not. Children who re‐experienced loneliness were less likely to be older, male, or Black, and more likely to belong to other racial or ethnic groups. Baseline loneliness emerged as an important correlate of prospective loneliness: at baseline, 12% of participants (*n* = 1,158) were reported by their parents to feel lonely. Among those, 71.5% (*n* = 829) continued to experience loneliness over the subsequent 3 years.

**Table 1:** Baseline characteristics of children with and without prospective loneliness

Characteristics	Sum of loneliness in follow‐ups			*p*‐Value^b^
	Overall	No	Yes	
	*N* = 9,602^a^	*N* = 7,098^a^	*N* = 2,504^a^	
Loneliness at baseline	1,158 (12%)	329 (4.6%)	829 (33%)	<.001
Age (months)	119.01 ± 7.52	119.18 ± 7.51	118.53 ± 7.52	<.001
Sex at birth				
Male	5,037 (52%)	3,856 (54%)	1,181 (47%)	<.001
Female	4,565 (48%)	3,242 (46%)	1,323 (53%)	
Race/Ethnicity				
White	5,317 (55%)	3,918 (55%)	1,399 (56%)	<.001
Black	1,305 (14%)	1,034 (15%)	271 (11%)	
Other	2,980 (31%)	2,146 (30%)	834 (33%)	

^a^ *n* (%); mean ± SD.

^b^ Pearson's chi‐squared test; Wilcoxon rank sum test.

### Familial and broader environmental correlates of prospective loneliness

**Figure 1:**
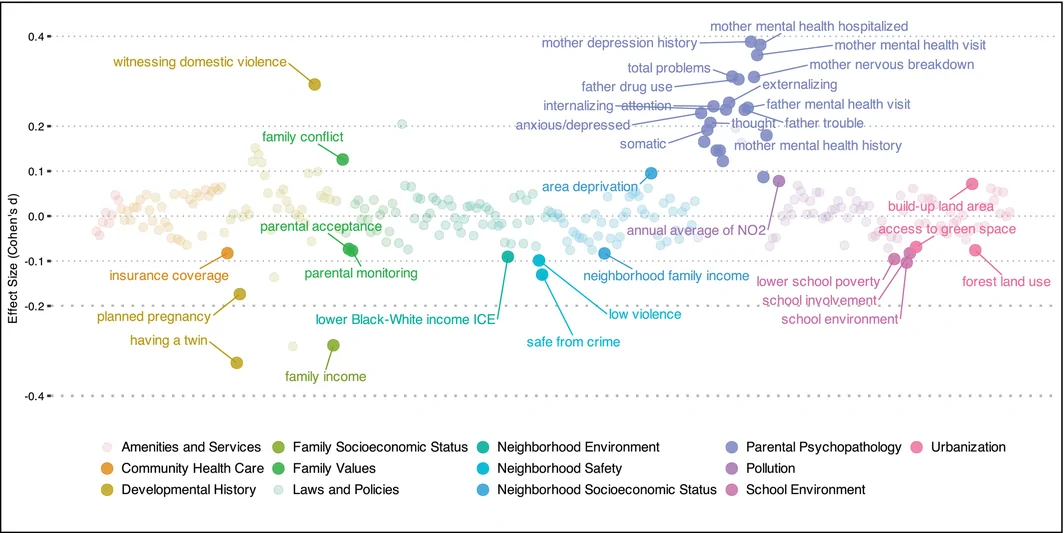
Associations between prospective loneliness and environmental variables across various domains. Of 294 environmental variables, 40 showed significant associations with loneliness after adjusting for multiple comparisons using a Bonferroni approach. The y‐axis represents the effect size (Cohen's *d*). Dimmed points indicate insignificant associations. Details of results are available in the Appendix S3

Our findings revealed predominantly small‐to‐moderate effect sizes for the observed associations between environments and prospective loneliness. Of the 294 environmental variables examined, 13.61% (*n* = 40) were significantly associated with prospective loneliness, after adjusting for baseline loneliness and demographic covariates (Figure 1 and Appendix S2). Parental psychopathology emerged as the strongest effect size with prospective loneliness, with effect sizes ranging from *d* = 0.087 to 0.388. Other prominent associations included having a twin (devhx_5_p, *d* = −0.327, 95% CI = [−0.428, −0.225]), witnessing domestic violence (ksads_ptsd_raw_766_p, *d* = 0.293, 95% CI = [0.159, 0.427]), family income (demo_comb_income_v2, *d* = −0.287, 95% CI = [−0.392, −0.182]), planned pregnancy (devhx_6_p, *d* = −0.174, 95% CI = [−0.251, −0.097]), safe from crime (neighborhood3r_p, *d* = −0.130, 95% CI = [−0.166, −0.095]), family conflict (fes_p_ss_fc, *d* = 0.126, 95% CI = [0.095, 0.157]), and school environment (srpf_y_ss_ses, *d* = −0.104, 95% CI = [−0.138, −0.069]). These findings underscore the relevance of both familial and broader environmental contexts to children's vulnerability to loneliness.

### Mental and physical health correlates of prospective loneliness

**Figure 2:**
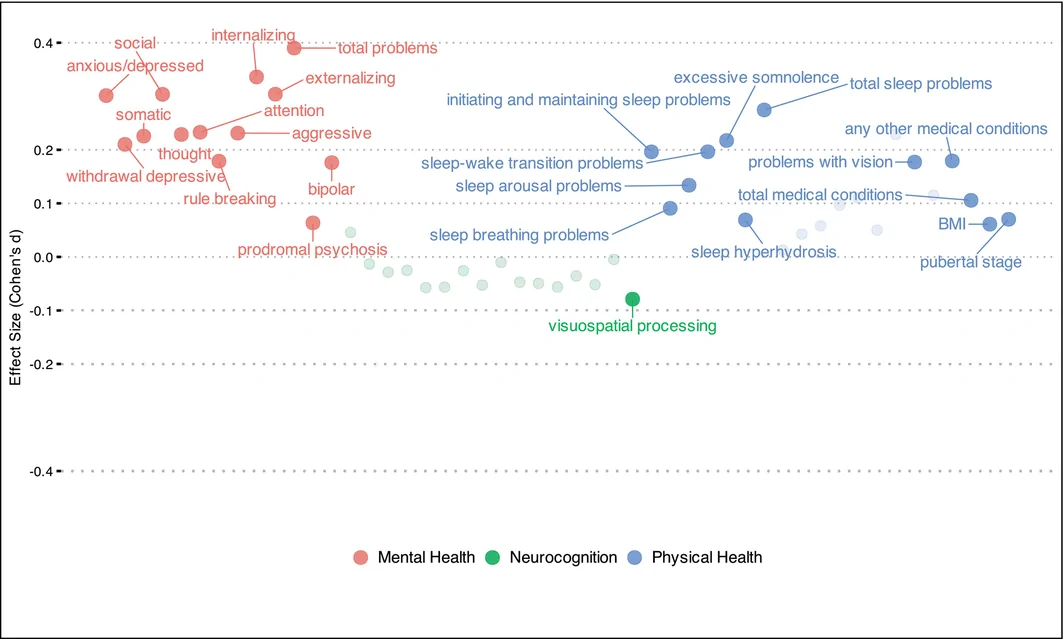
Prospective loneliness was linked to mental health, cognition, and physical health. Of 49 health measures, 26 showed significant associations with loneliness after adjusting for multiple comparisons using a Bonferroni approach. The *y*‐axis represents the effect size (Cohen's *d*). All the insignificant associations highlighted in gray were exclusively observed in cognition. Details of results are available in Appendix S4

We identified 26 out of 49 health variables (53.1%) that were significantly associated with prospective loneliness (Figure 2 and Appendix S2). All mental health variables were significantly associated with prospective loneliness, with effect sizes ranging from *d* = 0.070 to 0.390. General mental health problems showed the strongest associations, including total problems (cbcl_scr_syn_totprob_t, *d* = 0.390, 95% CI [0.354, 0.427]), internalizing problems (cbcl_scr_syn_internal_t, *d* = 0.336, 95% CI [0.297, 0.376]), and externalizing problems (cbcl_scr_syn_external_t, *d* = 0.304, 95% CI [0.265, 0.343]). Visuospatial processing was the only neurocognitive variable significantly associated with prospective loneliness (lmt_scr_efficiency, *d* = −0.079, 95% CI [−0.114, −0.044]). Among physical health variables, sleep problems emerged as the strongest association with prospective loneliness, with effect sizes ranging from *d* = 0.070 to 0.275. Medical conditions also showed significant associations (*d* = 0.106 to 0.179). Although smaller in magnitude, pubertal stage (*d* = 0.064, 95% CI [0.056, 0.101]) and BMI (*d* = 0.061, 95% CI [0.027, 0.095]) were also linked to prospective loneliness.

### Multimodal neural correlates of prospective loneliness

Because the primary aim of the present analysis was inferential rather than predictive, interpretation focused on multivariate contribution patterns rather than discriminative performance. Classification performance is reported in the Appendix S2 for transparency and completeness.

**Figure 3:**
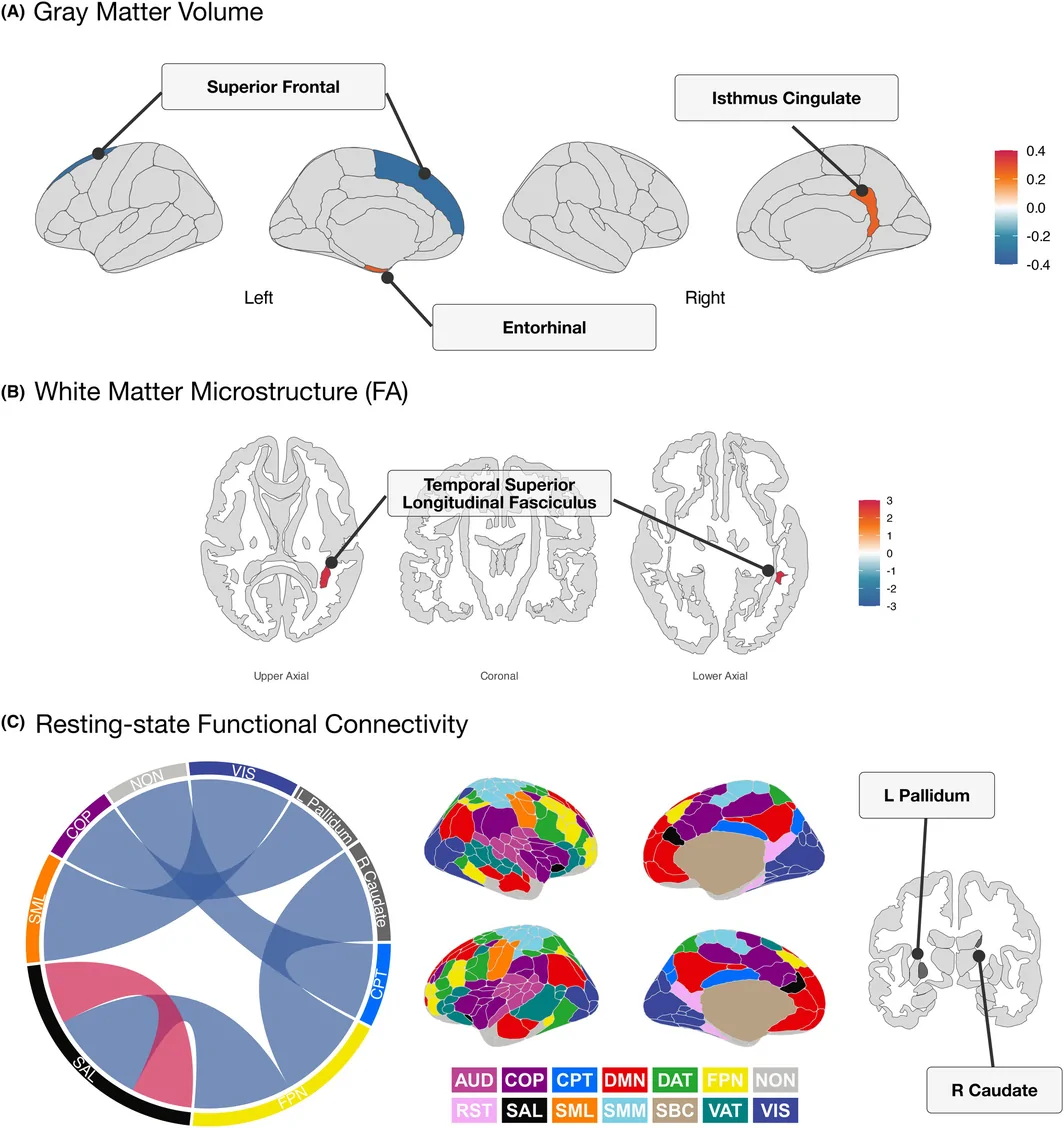
Linear discriminant analysis revealed distinct multimodal MRI profiles differentiating children with and without prospective loneliness. (A) Gray matter volumes contributing to group separation included the superior frontal gyrus, entorhinal cortex, and isthmus of the cingulate gyrus. (B) White matter microstructure analysis revealed significant contributions in the right temporal superior longitudinal fasciculus. (C) Altered connectivity patterns included reduced coupling between the frontoparietal and salience networks, the somatosensory and visual networks, and between the frontoparietal network and right caudate, as well as between the cingulo‐opercular network and left pallidum. Additionally, increased within‐network connectivity was observed in the salience network. Blue indicates negative associations, and red indicates positive associations

Structural MRI analyses revealed that individuals with prospective loneliness could be differentiated based on gray matter volume (GMV) in three cortical regions (Figure 3A and Appendix S2). Specifically, increased GMV in the left entorhinal cortex (weight_LDA_ = 0.262, 95% CI [0.063, 0.471]) and right isthmus cingulate cortex (weight_LDA_ = 0.254, 95% CI [0.041, 0.466]), and decreased GMV in the left superior frontal cortex (weight_LDA_ = −0.323, 95% CI [−0.615, −0.043]) contributed significantly to the classification of prospective loneliness status.

Diffusion MRI findings further supported the involvement of frontotemporal pathways (Figure 3B and Appendix S2). Children with prospective loneliness were characterized by greater fractional anisotropy in the right temporal superior longitudinal fasciculus (weight_LDA_ = 2.74, 95% CI [0.66, 5.32]). These results, consistent with the GMV findings, highlight spatial convergence in frontal and temporal regions associated with prospective loneliness.

Resting‐state functional connectivity analyses revealed several network‐level disruptions associated with prospective loneliness (Figure 3C and Appendix S2). Reduced connectivity was observed between the cingulo‐parietal and unlabeled networks (weight_LDA_ = −0.186, 95% CI = [−0.333, −0.030]), fronto‐parietal and salience networks (weight_LDA_ = −0.239, 95% CI = [−0.407, −0.061]), sensorimotor (mouth) and visual networks (weight_LDA_ = −0.246, 95% CI = [−0.447, −0.044]), cingulo‐opercular and left‐pallidum (weight_LDA_ = −0.173, 95% CI = [−0.333, −0.011]), and between the frontoparietal network and the right caudate (weight_LDA_ = −0.229, 95% CI = [−0.423, −0.035]). In contrast, increased within‐network connectivity was observed in the salience network (weight_LDA_ = 0.149, 95% CI = [0.026, 0.289]). These findings suggest that disruptions in large‐scale networks involved in cognitive control, salience detection, and sensorimotor integration may underlie vulnerability to future loneliness.

Sensitivity analyses further indicated moderate correspondence between LDA weights and mass‐univariate effect sizes for GMV (*r* = .56, *p* < .001) and weaker but significant correspondence for RSFC (*r* = .38, *p* < .001), supporting the interpretability of the multivariate contribution patterns (see Appendix S2). In contrast, WMM showed no such correspondence (*r* = .02, *p* = .86), suggesting limited consistency across analytic approaches. This pattern suggests that GMV and RSFC capture more stable and interpretable neurobiological signals, whereas the WMM findings may be less robust across analytic approaches.

### Sensitivity analyses demonstrating robustness

Across all sensitivity analyses, the overall pattern of findings remained consistent with the primary results. Correspondence between estimates from the primary and sensitivity analyses was quantified by correlating effect sizes (for mass‐univariate models) and LDA weights (for multivariate analyses) across domains, indicating that the identified associations were stable and not driven by specific analytic choices or sample composition. Detailed results are provided in the Appendix S2 and Figures S2–S5.

## Discussion

This study presents a novel investigation within a population‐based cohort of prospective loneliness in late childhood and early adolescence. Individual effect sizes for environmental and health variables were generally modest. However, the consistency of associations observed across diverse domains supports a multilevel risk model of loneliness (Bower et al., 2023; Lim, Eres, & Vasan, 2020; Marquez et al., 2023). Identifying consistent patterns across environmental, health, and neurobiological domains provides insight into their relative contributions to prospective loneliness. Our findings help prioritize candidate risk and protective factors that may inform future causal modeling, prevention strategies, and intervention development. Accordingly, interpretation emphasizes the overall pattern and magnitude of effect sizes, with particular attention to comparatively larger effects that may offer greater practical relevance. We highlight three key findings that guide our discussion. First, prospective loneliness was associated with a broad range of baseline environments, including parental psychopathology, developmental history, family dynamics, school environment, and neighborhood safety. Second, both physical and mental health indicators were significantly associated with prospective loneliness. Third, prospective loneliness was associated with alterations in brain structure and function within higher‐order cognitive networks involved in socioemotional processing, pointing to potential neurodevelopmental pathways of loneliness vulnerability.

Consistent with prior research, our findings suggest that prospective loneliness is associated with diverse psychosocial, familial, and contextual factors measured at baseline across ecological levels (Bower et al., 2023; Lim et al., 2020; Marquez et al., 2023). Late childhood is a critical developmental period marked by a shift from family‐centered to peer‐centered social interactions (De Goede, Branje, Delsing, & Meeus, 2009), while the brain undergoes extensive reorganization in areas subserving social cognition, self‐concept, and emotion regulation (Blakemore, 2008). Disruptions in the social or physical environment during this sensitive window may therefore carry amplified consequences for socioemotional development. Encouragingly, many of the factors associated with loneliness identified in this study appear to be modifiable.

Among the baseline environmental variables examined, parental psychopathology emerged as the strongest candidate risk factor for prospective loneliness. This finding is consistent with prior longitudinal research showing that maternal depressive symptoms are associated with increased loneliness in offspring (Psychogiou et al., 2022). Our analyses extended this by demonstrating consistent associations across multiple dimensions of parental mental health, underscoring its broad and potent association on children's socioemotional development. While the origins of parental psychopathology likely reflect both genetic and environmental influences, its associations with loneliness may be partially mediated by family functioning (Shalev et al., 2019). Reduced parental responsiveness, heightened family conflict, or exposure to domestic violence may undermine children's sense of safety and belonging, disrupting the development of secure attachment and trust and increasing their vulnerability to social disconnection. Interventions aimed at improving parental mental health and strengthening family support systems may offer effective avenues for mitigating loneliness risk. Future studies should explore family functioning as a potential mediator linking parental psychopathology to children's social well‐being.

Enhancing protective school‐level factors, such as school climate and student involvement, may also help buffer against loneliness. A large‐scale study spanning 23 European countries found that students in schools with more bullying and less cooperative climates were significantly more likely to report loneliness (Schnepf et al., 2023). Notably, school characteristics accounted for more variance in loneliness than individual demographic variables. Our findings, consistent with this research, highlight the role of schools not only as educational settings but also as critical environments for social development. Investing in inclusive, supportive school climates could thus be a key strategy for loneliness prevention.

Our results further emphasize the importance of the built environment, which can be distinguished between ‘structured’ components (e.g., housing density, open space, green space) and ‘lived’ components (e.g., perceived safety, affordability) (Bower et al., 2023). While both types were associated with loneliness, our findings suggest that lived environmental features, especially neighborhood safety, were more strongly associated with prospective loneliness than structured ones. These findings point to the psychological salience of environmental perceptions: feeling unsafe or unstable in one's surroundings may be associated with greater social withdrawal in children, reinforcing feelings of loneliness. Community‐level interventions that enhance neighborhood safety, such as increased community policing or fairer law enforcement practices, may therefore be particularly effective in reducing childhood loneliness (Corman & Mocan, 2005; Sousa & Kelling, 2001).

Existing literature has already documented the bidirectional relationships between mental health and loneliness (Hards et al., 2022). A meta‐analysis showed similar effect sizes of cross‐lagged panels between depressive symptoms and loneliness (Chen, Song, Lee, & Zhang, 2023). Evidence also suggests that loneliness may precede psychiatric disorders, with lonely individuals at a higher risk for new onset depression (Mann et al., 2022) and schizophrenia spectrum disorder (Andreu‐Bernabeu et al., 2025). The long‐term impact of loneliness is evident as childhood loneliness has been linked to anxiety and depression in young adults (Xerxa, Rescorla, Shanahan, Tiemeier, & Copeland, 2023). Our study revealed that the effect sizes for associations between prospective loneliness and baseline mental health were comparable to those for parental psychopathology. Thus, considering the intergenerational transmission of psychopathology between parents and children (Branje et al., 2020) could offer a potential solution to break this vicious cycle. Additionally, similar to mental health, sleep quality and medical problems demonstrated significant but modest effect sizes associated with prospective loneliness, in line with the literature (Matthews et al., 2017). Poorer physical health might reduce physical activity, subsequently limiting social interactions with peers (Di Bartolomeo & Papa, 2019). Lastly, the interplay between mental health, physical health, and loneliness requires further investigation (Lau et al., 2025).

We further investigated the neurobiological profiles of prospective loneliness in children across structural, diffusion, and functional imaging modalities. Structurally, the superior frontal gyrus, entorhinal cortex, and isthmus of the cingulate gyrus were associated with prospective loneliness in children. Prior studies have shown that gray matter density in the entorhinal cortex correlates with social network size (Kanai, Bahrami, Roylance, & Rees, 2012). The superior frontal gyrus forms part of the medial prefrontal cortex (mPFC), a region consistently associated with loneliness in prior research (Lam et al., 2021). The mPFC and the posterior cingulate cortex, which includes the isthmus of the cingulate gyrus, are core midline components of the default mode network (DMN) (Menon, 2023; Raichle et al., 2001). These regions are critically involved in internally directed processes such as self‐referential thinking and mentalizing, functions that are closely tied to social connection (Courtney & Meyer, 2020). Disruptions in these processes may impair social cognition and increase vulnerability to loneliness (Spreng et al., 2020). Notably, the isthmus of the cingulate has also been associated with depression (Schmaal et al., 2017) and childhood trauma (Jeong et al., 2021), suggesting it may reflect broader neurodevelopmental sensitivity to early adversity. These findings point to a frontotemporal pathway through which early life experiences may be associated with the emergence of loneliness and related emotional difficulties.

Diffusion MRI findings further reinforce this frontotemporal interpretation. Specifically, reduced integrity was observed in the right temporal superior longitudinal fasciculus (SLF), a major association tract that links temporal regions to the prefrontal cortex (Hagler Jr et al., 2009). The SLF has been implicated in language processing (Dick & Tremblay, 2012) and, more recently, has been associated with social health outcomes, including loneliness (Costanzo et al., 2023). Together with the gray matter findings, these results suggest that prospective loneliness may be associated with altered frontotemporal structural connectivity, potentially compromising the integration of social information and self‐other representations essential for socioemotional development.

While our structural findings may highlight gray matter volume differences in regions overlapping with DMN hubs, our resting‐state functional connectivity (RSFC) analyses did not reveal significant involvement of DMN. Instead, RSFC alterations were observed in circuits involving the frontoparietal, salience, sensorimotor, and subcortical networks. This divergence may reflect developmental differences in how loneliness is functionally instantiated in the brain. In children, loneliness may manifest more strongly through disruptions in attentional, emotional salience, and sensorimotor integration networks, rather than in internally directed processes typically associated with the DMN in adults (Spreng et al., 2020). This aligns with evidence that the frontoparietal and salience networks play key roles in emotion regulation and the dynamic allocation of cognitive and affective resources during social interactions (Menon, 2011; Seeley et al., 2007). Disruptions in these networks may impair children's ability to regulate emotions and process social feedback adaptively, increasing their vulnerability to psychopathology (Menon, 2011). In turn, these difficulties may elevate the risk of experiencing prospective loneliness, as suggested by our findings.

Reducing loneliness can enhance well‐being, reduce risk for later mental health problems, and promote overall health and longevity in society (Jeste, Lee, & Cacioppo, 2020). The present findings highlight several near‐term, potentially actionable targets across multilevel environmental systems. At the family level, early screening during pregnancy for parental psychopathology may help reduce developmental risk related to children's socioemotional functioning (Earls et al., 2019). At the school level, monitoring school climate, bullying exposure, and student connectedness may offer opportunities for early identification, while interventions that promote inclusive peer environments and student engagement may help buffer loneliness risk (Schnepf et al., 2023). At the community level, indicators such as perceived neighborhood safety may support risk surveillance and help identify vulnerable contexts, while broader societal strategies, including family and reproductive health supports, may also play a preventive role (Finer & Zolna, 2016). Given the multifactorial nature of loneliness, developmentally sensitive and multilevel approaches spanning family, school, and community contexts may be most appropriate (Mann et al., 2017), complementing individual‐level interventions (Hawkley, 2022; Pitman, Mann, & Johnson, 2018).

However, current intervention evidence for loneliness remains limited. Although a recent meta‐analysis found that youth interventions were generally effective, most focused on mitigating mental health risks rather than directly targeting loneliness (Eccles & Qualter, 2021). A more recent meta‐analysis suggests that loneliness‐focused interventions in children and adolescents may yield small reductions in loneliness, primarily when they incorporate social and emotional learning (Burke et al., 2026). These findings are broadly consistent with our neuroimaging findings implicating brain systems involved in socioemotional processing. Together, this convergence highlights the potential relevance of socioemotional processes as targets for intervention, while noting that these interpretations remain associative rather than mechanistic.

At the same time, these implications should be interpreted cautiously. The present findings are associative and do not establish causal effects or intervention efficacy. Translating these results into intervention design will require additional evidence, including longitudinal and mechanistic studies to identify modifiable mediators, such as family functioning and social connectedness, as well as randomized control trials to test whether changing these correlates can reduce prospective loneliness.

Several limitations of the current study should be noted. First, loneliness is a subjective and multifaceted experience. The ABCD study was not designed to assess loneliness in a targeted or comprehensive manner; accordingly, the parent‐reported CBCL item represents the closest available indicator in this cohort. As a single dichotomized measure, it may not fully capture the multifaceted nature of loneliness (Astell‐Burt et al., 2022), may have lower reliability, and may be more susceptible to measurement error than validated multi‐item instruments. This item may also capture related but distinct phenomena, such as social withdrawal or negative affect. Accordingly, the present findings should be interpreted as reflecting a coarse indicator of parent‐perceived loneliness‐related experience. In addition, parent reports may not fully reflect children's subjective experiences and may introduce informant‐specific bias. As no comparable child‐reported measure of loneliness is available, informant effects (parent vs. child) cannot be examined, limiting our ability to assess potential discrepancies between parent perceptions and children's subjective experiences. Its dichotomization may further reduce statistical power and obscure subclinical variation (Altman & Royston, 2006). The binary ‘any follow‐up’ definition was adopted to provide a stable and interpretable outcome aligned with the study aim of identifying children at elevated risk of prospective loneliness, rather than modeling severity gradients or longitudinal trajectories. Thus, the present approach does not distinguish between transient, recurrent, or persistent loneliness and may obscure heterogeneity in developmental trajectories. Nevertheless, the ABCD study provides a unique opportunity to examine prospective loneliness in relation to a broad range of health, environmental, and neurobiological measures within the same population‐based cohort, offering a valuable framework for understanding multilevel correlates of prospective loneliness. Future research should incorporate validated measures, such as the UCLA Loneliness Scale (De Jong Gierveld & van Tilburg, 2006), to examine symptom severity and developmental trajectories more directly. Second, several measures examined in this study were based on a parent report, which raises the possibility of common method bias (Podsakoff, MacKenzie, Lee, & Podsakoff, 2003; Podsakoff, MacKenzie, & Podsakoff, 2012). In particular, parental stress or burden may have influenced perceptions of children's loneliness, sleep, and emotional or behavioral difficulties, potentially inflating associations among these variables. At the same time, given the young age of the cohort at baseline, a parent report was a practical and developmentally appropriate method for collecting broad behavioral and health information in a large population‐based study. Nevertheless, this issue is especially relevant when interpreting associations among parent‐reported measures and should be considered when evaluating the strength and specificity of the observed findings. Third, follow‐up assessments in the ABCD cohort spanned pre‐pandemic, pandemic, and post‐pandemic periods, and pandemic‐related disruptions may have influenced loneliness reports. Although our sensitivity analyses showed that the overall pattern of results remained highly similar after restricting the first loneliness report to the pre‐pandemic period, this context should still be considered when interpreting prospective loneliness in the cohort. Fourth, our current univariate analytic approach may not fully capture the complex interplay among factors associated with loneliness. This approach may overlook synergistic effects, moderating relationships, and residual confounding by unmeasured variables. Accordingly, some observed associations may partly reflect omitted behavioral, clinical, or contextual factors. Nevertheless, the present findings may help guide the selection of candidate risk and protective factors for future multivariate and longitudinal research. Fifth, although the ABCD cohort represents a diverse American sample in terms of race and ethnicity, external validation is still necessary. In particular, cultural factors may shape how loneliness is perceived, reported, and experienced. While some studies suggest that loneliness may be reported more frequently in individualistic cultures, its psychological consequences may be more detrimental in collectivist cultures, where social connectedness is more strongly emphasized and deviations from group integration may carry greater social and psychological costs (Barreto et al., 2021). Sixth, the mass‐univariate screening approach involves a large number of correlates, and the use of Bonferroni correction, while providing stringent control of the family‐wise error rate, may be overly conservative and reduce sensitivity to smaller but potentially meaningful associations. As a result, some true effects may have been missed. Although we emphasize the overall pattern and magnitude of effect sizes rather than statistical significance alone, the present findings should be interpreted as a conservative set of associations. Future studies may complement this approach with alternative multiple‐testing procedures, such as false discovery rate control, to better balance sensitivity and specificity. Finally, the current study used a predefined atlas for brain analysis, which might overlook small and localized effects of the brain on loneliness. Future research employing voxel‐wise analysis could identify more localized disruptions in the brain associated with loneliness.

In conclusion, this systematic and comprehensive investigation revealed the relationships of child prospective loneliness with environment, health, and brain. The findings support early interventions that promote healthy socioemotional development by addressing parental mental health, neighborhood safety, and school climate. Importantly, our results underscore the need for a multilevel, personalized approach that integrates biological, individual, environmental, and societal factors to effectively address the increasing challenge of loneliness in children.

## Ethical considerations

The Adolescent Brain Cognitive Development ABCD Study was approved by the institutional review board of the University of California, San Diego (IRB# 160091, approved September 13, 2016), as well as the institutional review boards of each of the 21 data collection sites. Written informed consent was obtained from all parents or legal guardians, and written informed assent was obtained from all child participants.

**Key points**

**What's known?**

Childhood loneliness predicts poorer mental and physical health, but early candidate multilevel risk and protective factors are not fully characterized.

**What's new?**

In a population cohort of 9,602 ABCD participants, loneliness at ages 11–13 was prospectively associated with 40 environmental, 26 health, and multiple neuroimaging indicators measured at ages 9–10.

**What's relevant?**

Strongest candidate risk factors involved parental psychopathology, developmental history, child mental health, and socioemotional brain systems, highlighting modifiable family, school, and neighborhood conditions for early identification and prevention.

## Supporting information


**Appendix S1.** Supplementary methods.
**Appendix S2.** Supplementary results.
**Table S1.** Sample characteristics of included vs. excluded participants.
**Figure S1.** Flowchart of participant inclusion and exclusion.
**Figure S2.** Validation of LDA‐derived feature weights via correspondence with mass‐univariate effect sizes.
**Figure S3.** Correspondence of effect estimates between the primary analysis and sensitivity analysis restricted to first‐year loneliness across domains.
**Figure S4.** Correspondence of effect estimates between the primary analysis and sensitivity analysis restricted to no baseline (incident) loneliness across domains.
**Figure S5.** Correspondence of effect estimates between the primary and pre‐pandemic sensitivity analyses across domains.

**Appendix S3.** Variable sheet.

**Appendix S4.** Result sheet.

## Data Availability

The data that support the findings of this study are openly available in the NIMH Data Archive (NDA) at https://abcdstudy.org, reference number https://doi.org/10.15154/z563‐zd24.
