# Supplementary material for: What makes a lonely child: environmental, health, and multimodal neuroimaging correlates of prospective loneliness in the ABCD study
Source: J Child Psychol Psychiatry. 2026 May 25;67(10):1714–28. doi: 10.1111/jcpp.70178 (PMC13535708; doi:10.1111/jcpp.70178)
Supplement: Supplementary file 1 [file JCPP-67-1714-s002.docx]

**What makes a lonely child: Environmental, health, and multimodal neuroimaging correlates of prospective loneliness in the ABCD study**

**Supporting Information**

**Appendix S1. Supplementary Methods**

***Selection Bias Assessment***

**Table S1** compares demographic characteristics between included and excluded participants to evaluate potential selection bias arising from the exclusion criteria. No significant differences were observed for sex (*q* = 0.30) or age (*q* = 0.40), suggesting that the included and excluded groups were broadly comparable on these basic demographic characteristics. Baseline loneliness was slightly more frequent among excluded than included participants (14% vs. 12%), but this difference did not survive multiple-comparison correction (*q* = 0.091). In contrast, race/ethnicity differed significantly between groups (*q* < 0.001), with the included sample containing a higher proportion of White participants and a lower proportion of Black participants than the excluded sample. These findings suggest some demographic imbalance related to sample inclusion, which should be considered when interpreting the generalizability of the results.

***Non-imaging Variables used in the Current Study***

Details of each included variable are available in the ***Appendix S3****.* In brief, we have included 347 environmental variables across 13 categories, including Amenities and Services, Community Health Care, Developmental History, Family Values, Laws and Policies, Neighborhood Environment, Neighborhood Safety, Neighborhood Socioeconomic Status, Parental Psychopathology, Pollution, School Environment, Family Socioeconomic Status, and Urbanization. Health measures comprised 61 indicators, encompassing mental health, neurocognition, and physical health, including anthropometric measurements, sleep disturbances, medical history, and pubertal development. Mental health was evaluated through CBCL dimensions, psychotic-like experiences, and subsyndromal mania symptoms. Neurocognition was assessed with NIH Toolbox^®^ tests, Little Man Test, WISC-V Matrix Reasoning, and RAVLT. Physical health encompassed BMI, waist measurements, sleep disturbances, medical history, and pubertal development, providing a comprehensive overview of children's well-being.

***Neuroimaging Variables***

All the data used for analysis in this study were preprocessed by the ABCD team and were downloaded in December 2023 as part of the 5.1 release (DOI: 10.15154/z563-zd24). The ABCD study employed standardized acquisition, preprocessing and analysis protocols which have been described in detail (Casey et al., 2018; Garavan et al., 2018; Hagler et al., 2019) [^1–3^](https://paperpile.com/c/bEBB2r/feioN+nwpod+6sNo5). We outline the neuroimaging acquisition and analysis protocols implemented by the ABCD study relevant to the analyses presented here.

***Structural Magnetic Resonance Imaging (sMRI)***

During the acquisition of 3D T1-weighted images, a child-friendly movie was played. The 3D T1-weighted magnetization-prepared rapid acquisition gradient echo (MPRAGE) scan was performed for cortical parcellation and subcortical segmentation with the following parameters: image matrix = 256x256, voxel resolution = 1x1x1 mm³ isotropic, repetition time (TR) = 2500 ms, echo time (TE) = 2.88 ms, field of view (FOV) = 256x256 mm, and flip angle = 8 degrees. Prospective motion correction (PROMO) was implemented on General Electric (GE) systems, and Volumetric Navigators (vNav) were used for prospective motion correction and selective reacquisition on Siemens and Philips platforms.

Cortical reconstruction and volumetric segmentation of structural datasets were performed by the ABCD Data Analysis, Informatics, and Resources Center (DAIRC) using the FreeSurfer image analysis suite (version v7.1.1), which is well-documented and freely available (<http://surfer.nmr.mgh.harvard.edu/>). The processing steps included the removal of non-brain tissue using a hybrid watershed/surface deformation procedure, automated Talairach transformation, segmentation of subcortical white matter and deep gray matter structures, intensity normalization, tessellation of the gray/white matter boundary, automated topology correction, and surface deformation based on intensity gradients to accurately define the gray/white and gray/cerebrospinal fluid borders. This method incorporates both intensity and continuity information from the entire three-dimensional MR volume to produce representations of cortical thickness, which is calculated as the closest distance from the gray/white boundary to the gray/CSF boundary at each vertex on the tessellated surface. This process yielded 68 cortical and 14 subcortical volumetric measures.

***Diffusion Magnetic Resonance Imaging (dMRI)***

The diffusion magnetic resonance imaging (dMRI) data were acquired using multiband EPI with a slice acceleration factor of 3, across 96 diffusion directions at 1.7 mm isotropic resolution, including seven b=0 frames and four b-values (6 directions with b=500 s/mm², 15 directions with b=1000 s/mm², 15 directions with b=2000 s/mm², and 60 directions with b=3000 s/mm²). Each acquisition block included fieldmap scans for B0 distortion correction.

Eddy current correction (ECC) used a model-based approach, predicting distortions based on diffusion gradient orientations and amplitudes, focusing on displacement along the phase-encoded direction. A robust tensor fit excluded dark slices caused by abrupt head motion to prevent these slices from influencing ECC. RMS error for each frame was normalized, and frames with an RMS greater than 3.2 were censored. This process was iterated three times to exclude dark frames, which were replaced with synthesized images from the censored tensor fit.

ECC was optimized using Newton's method, minimizing RMS error between eddy-current-corrected images and synthesized images from the censored tensor fit. The corrected images were rigid-body-registered to the synthesized volume from the post-ECC censored tensor fit, and the diffusion gradient matrix was adjusted for head rotation. Spatial and intensity distortions from B0 field inhomogeneity were minimized using a fast, nonlinear registration procedure that aligned pairs of b=0 images with opposite phase-encoding polarities. Gradient nonlinearity distortions were corrected, and b=0 dMRI images were registered to T1-weighted structural images using mutual information, resampled at 1.7 mm isotropic resolution.

Major white matter tracts were labeled using AtlasTrack, a probabilistic atlas-based method for segmenting white matter fiber tracts. Structural MRI images were nonlinearly registered to the atlas using discrete cosine transforms (DCT), and diffusion tensor imaging (DTI)-derived orientations were compared to the atlas fiber orientations to refine tract location probabilities. FreeSurfer's automated brain segmentation excluded gray matter and cerebrospinal fluid voxels. DTI measures such as fractional anisotropy (FA), mean axial diffusivity (AD), and radial diffusivity (RD) were calculated using a standard linear estimation approach with log-transformed diffusion-weighted signals, and mean DTI measures were calculated for white matter tract ROIs created with AtlasTrack, using probability estimates for weighted averages of DTI measures.

***Resting-State Functional Magnetic Resonance Imaging (rs-fMRI)***

Resting-state functional MRI (rsfMRI) data were acquired using a multiband echo-planar imaging (EPI) sequence with a slice acceleration factor of 6, a TR of 800 ms, and 2.4 mm isotropic resolution. Each acquisition block included field map scans for B0 distortion correction. Head motion was corrected by registering each frame to the first using AFNI’s *3dvolreg*, which also generated motion time courses used in single-subject analyses. B0 distortions were addressed using the reversing polarity method. To minimize signal dropout from within-voxel field gradients in gradient-echo images, displacement fields were estimated from spin-echo calibration scans, adjusted for between-scan motion, and applied to the gradient-echo series.

Images were subsequently corrected for gradient nonlinearity distortions. To address between-scan motion, each scan was resampled using cubic interpolation and aligned to a reference scan (selected near the temporal midpoint) based on rigid-body registration of the first frame of each scan. Automated registration between spin-echo field maps and T1-weighted structural images was performed using mutual information, with coarse alignment based on atlas-based within-modality registration. The final fMRI data remained in native space with 2.4 mm isotropic resolution.

Functional connectivity was computed using a seed-based, correlation approach adapted for surface-based analysis. Pre-analysis steps (performed after initial fMRI preprocessing) included removal of initial frames, intensity normalization, nuisance regression, temporal filtering, and extraction of average time series for each region of interest (ROI). Time series normalization was done by dividing each voxel's signal by its mean across time. Nuisance regression removed quadratic trends, motion parameters (six directions plus derivatives and squares), and the averaged time courses of white matter, ventricles, and whole brain, along with their first derivatives. These nuisance ROIs were defined using FreeSurfer’s automated segmentation (aseg), resampled to fMRI space, and eroded by one voxel.

Pairwise correlations between ROIs were calculated, Fisher z-transformed, and averaged within or between functional networks (defined by the Gordon parcellation). Within-network connectivity was computed as the average of all unique ROI pair correlations within the same network. Between-network connectivity was computed similarly by averaging correlations across all ROI pairs spanning two different networks.

***Selection of Participants for Analyses***

**Figure S1** illustrates the inclusion and exclusion process for participants. Data release 5.1 included 11,868 children at baseline. We then removed participants without reported sex at birth, without participant ID, site ID, age, sex, race, and/or loneliness data collected from baseline, year 1, year 2, and year 3 follow-ups (n = 2,266). For environment variables, 132 participants were further excluded because they had over 15% missing values. For health variables, 116 participants were further excluded because they had over 15% missing values.

For gray matter volume analyses, we excluded participants whose scans did not pass quality assurance (n = 870). Two quality assurance measures were used: the MRI Report Score (mrif_score) and inclusion criteria for T1-weighted (T1w) imaging (imgincl_t1w_include = 1). We included participants with mrif_score categories 1 or 2, indicating no abnormal findings or normal anatomical variants of no clinical significance. Participants included in the T1w analyses met the following criteria: T1w series passed raw quality control, FreeSurfer QC not failed, and derived results exist.

For dMRI analyses, we excluded participants whose scans did not pass quality assurance (n =1,650). The dMRI (DTI/RSI) series meets all criteria for inclusion (imgincl_dmri_include = 1). The criteria for inclusion are the dMRI series must pass raw quality control and have at least 103 repetitions, or if the manufacturer is Philips, at least 2 series and 51 repetitions. The T1 series must pass raw quality control. The dMRI B0 unwarp must be available. FreeSurfer QC must not fail, and the dMRI manual post-processing QC must not fail. The dMRI registration to T1w must be below 17. The dMRI dorsal cutoff score must be below 47, and the ventral cutoff score must be below 54. Finally, derived results must exist.

For rs-fMRI analyses, we excluded participants whose scans did not pass quality assurance (n =2,258). The rs-fMRI series were included if they met all quality control (QC) criteria (indicated by mr_qc_incl_rsfmri_indicator = 1). These criteria included: successful raw QC for both rsfMRI and T1-weighted scans, more than 375 frames remaining after motion censoring, availability of B0 unwarping data, no failure in FreeSurfer or manual post-processing QC, acceptable fMRI-to-T1w registration (rigid score < 19), and acceptable dorsal and ventral field-of-view cutoff scores (< 65 and < 60, respectively). Additionally, derived connectivity results must be available and not missing.

Further details on MRI quality control procedures are available at: <https://docs.abcdstudy.org/latest/documentation/imaging/type_qc.html>.

***Excluding Potential COVID-19 Pandemic Effects on Loneliness***

Because part of the follow-up period overlapped with the COVID-19 pandemic, we further classified prospective loneliness reports according to interview date into pre-pandemic (before March 2020), pandemic (March 2020 to July 2021), and post-pandemic (after July 2021) periods (Gatavinš et al., 2025) for sensitivity analyses, in order to evaluate whether the primary findings were robust to pandemic-related disruptions that may have influenced loneliness reports.

***Sensitivity Analysis***

To evaluate the robustness of the findings, we conducted three additional sensitivity analyses. First, to assess temporal stability and whether the observed associations were driven by longer-term aggregation across follow-up waves, we repeated all analyses using loneliness at the 1-year follow-up as the outcome. Second, to isolate incident cases and reduce potential confounding by pre-existing loneliness, we repeated the analyses in the subsample of participants without baseline loneliness. Third, to evaluate the potential influence of the COVID-19 pandemic on prospective loneliness (Farrell et al., 2023) , we restricted the sample to participants whose first loneliness report occurred before the pandemic period. For each sensitivity analysis, we repeated the full analytic pipeline for both the mass-univariate and LDA analyses. Robustness was assessed by correlating estimates from the primary and sensitivity analyses across domains, using effect sizes for the mass-univariate analyses and LDA weights for the multivariate analyses.

**Appendix S2. Supplementary Results**

Details of result tables are available in the ***Appendix S4***. For environment and health analysis, “Cohen's d” measures the effect sizes converted from the regression models. The “95% CI (lower)” and “95% CI (upper)” columns provide the lower and upper bounds of the 95% confidence interval for “Cohen's d”, indicating the range within which the true effect size is likely to fall. The “p” column shows the p-value from the statistical test, indicating the probability that the observed results occurred by chance, while “p.adj” presents the adjusted p-value using Bonferroni correction, accounting for multiple comparisons to reduce the likelihood of false positives. For MRI analyses, the “LDA weight” column represents the mean linear discriminant analysis weight from 1,000 iterations, with associated 95% confidence intervals. A “significant” column indicates whether the confidence interval excludes zero. The columns include “variable_name”, which indicates the name of the variable based on the ABCD study being analyzed. The rest of the columns provide further details of the “variable_name”, including labels and specific details about the variable.

***LDA Classification Performance***

Because our primary aim was inferential rather than predictive, classification performance should not be taken as the main criterion for evaluating the LDA results. In this context, subtle and distributed neuroimaging effects can still yield interpretable multivariate contribution patterns even when discrimination is weak at the individual level (Kopal et al., 2023; Zhou et al., 2025). For transparency and completeness, we report classification performance for each modality. Performance was only slightly above chance for GMV and WMM (GMV: balanced accuracy = 0.551 ± 0.006, ROC AUC = 0.574 ± 0.005; WMM: balanced accuracy = 0.551 ± 0.006, ROC AUC = 0.572 ± 0.006), indicating limited individual-level discrimination. RSFC showed somewhat higher performance (balanced (balanced accuracy = 0.627 ± 0.007, ROC AUC = 0.676 ± 0.006), although this may partly reflect the greater dimensionality of the RSFC feature space, which included 338 connectivity edges. Together, these results indicate that neurobiological correlates of prospective loneliness are distributed and low in magnitude, yielding consistent multivariate contribution patterns at the group level but limited discriminability at the individual level. Accordingly, despite some separation in multivariate feature space, the lack of out-of-sample evaluation means that no conclusion can be drawn about predictive utility.

***Sensitivity Analyses*** ***to Evaluate Temporal Stability***

We examined whether the pattern of associations identified in the primary analysis was preserved when the outcome was restricted to loneliness at the 1-year follow-up. By focusing on the earliest follow-up window, this analysis isolated early-onset cases and provided an initial assessment of whether the observed associations were specific to a particular follow-up period (**Figure S3**). Across domains, effect estimates from the primary and sensitivity analyses were positively correlated (*p* < 0.001; environment: *r* = 0.66; health: *r* = 0.69; GMV: *r* = 0.53; RSFC: *r* = 0.46), indicating moderate-to-strong consistency in the overall pattern of findings. The correlation was weaker for WMM (*r* = 0.32), suggesting greater temporal variability or lower signal stability for this modality. Overall, these findings suggest that the primary results were largely robust to variation in follow-up window, although temporal stability appeared weaker for WMM.

***Sensitivity Analyses*** ***to Isolate Incident Loneliness***

We examined whether the pattern of associations identified in the primary analysis was preserved when restricting the sample to participants without baseline loneliness. By isolating incident cases, this analysis evaluated whether the observed associations were driven primarily by persistent or pre-existing loneliness at baseline (**Figure S4**). Across domains, effect estimates from the primary and sensitivity analyses were strongly correlated (p < 0.001; environment: r = 0.74; health: r = 0.85; GMV: r = 0.93; WMM: r = 0.79; RSFC: r = 0.89), indicating high consistency in the overall pattern of findings. These results suggest that the primary associations were not solely driven by participants with baseline loneliness and were largely preserved when analyses were restricted to incident cases.

***Sensitivity Analyses to Exclude Potential Pandemic Effects on Loneliness***

Because follow-up assessments in the ABCD cohort spanned pre-pandemic, pandemic, and post-pandemic periods, sensitivity analyses were conducted to evaluate whether the primary findings were influenced by COVID-19-related disruptions. Based on the timing of the first follow-up report of loneliness, 7,098 participants never reported loneliness, 1,662 first reported loneliness during the pre-pandemic period, 766 during the pandemic period, and 76 during the post-pandemic period. We then repeated all analyses including only participants whose first follow-up report of loneliness occurred during the pre-pandemic period, thereby focusing on prospective loneliness emerging before major pandemic-related disruptions in schooling, peer contact, and daily routines. The overall pattern of findings remained highly similar to that of the primary analyses. Correlations between parameter estimates from the primary and sensitivity analyses were strong across domains (**Figure S5**; all ps < 0.001), including environmental factors (*r* = 0.86), health indicators (*r* = 0.99), gray matter volume (*r* = 0.82), white matter microstructure (*r* = 0.70), and resting-state functional connectivity (*r* = 0.81). These findings indicate that the main results were largely robust to potential pandemic-related influences on loneliness reports.

**Table S1: Sample Characteristics of Included vs. Excluded Participants**

| **Characteristic** | **Excluded  N = 2,096^1^** | **Included  N = 9,602^1^** | **p-value^2^** | **q-value^3^** |
| --- | --- | --- | --- | --- |
| Sex at birth |  |  | 0.2 | 0.3 |
| Male | 1,068 (51%) | 5,037 (52%) |  |  |
| Female | 1,028 (49%) | 4,565 (48%) |  |  |
| Age (months) | 118 (112, 125) | 119 (112, 126) | 0.4 | 0.4 |
| Race/Ethnicity |  |  | <0.001 | <0.001 |
| White | 790 (38%) | 5,317 (55%) |  |  |
| Black | 564 (27%) | 1,305 (14%) |  |  |
| Other | 742 (35%) | 2,980 (31%) |  |  |
| Loneliness at baseline | 286 (14%) | 1,158 (12%) | 0.046 | 0.091 |
| ^1^n (%); Median (Q1, Q3) | | | | |
| ^2^Pearson's Chi-squared test; Wilcoxon rank sum test | | | | |
| ^3^False discovery rate correction for multiple testing | | | | |

**Figure S1. Flowchart of participant inclusion and exclusion.**

**Figure S2. Validation of LDA-derived feature weights via correspondence with mass-univariate effect sizes**

**Figure S3. Correspondence of effect estimates between the primary analysis and sensitivity analysis restricted to first-year loneliness across domains**

**Figure S4. Correspondence of effect estimates between the primary analysis and sensitivity analysis restricted to no baseline (incident) loneliness across domains**

**Figure S5. Correspondence of effect estimates between the primary and pre-pandemic sensitivity analyses across domains**

**References**

Casey, B. J., Cannonier, T., Conley, M. I., Cohen, A. O., Barch, D. M., Heitzeg, M. M., Soules, M. E., Teslovich, T., Dellarco, D. V., Garavan, H., Orr, C. A., Wager, T. D., Banich, M. T., Speer, N. K., Sutherland, M. T., Riedel, M. C., Dick, A. S., Bjork, J. M., Thomas, K. M., … ABCD Imaging Acquisition Workgroup. (2018). The Adolescent Brain Cognitive Development (ABCD) study: Imaging acquisition across 21 sites. *Developmental Cognitive Neuroscience*, *32*, 43–54. https://doi.org/10.1016/j.dcn.2018.03.001

Farrell, A. H., Vitoroulis, I., Eriksson, M., & Vaillancourt, T. (2023). Loneliness and well-being in children and adolescents during the COVID-19 pandemic: A systematic review. *Children*, *10*(2). https://doi.org/10.3390/children10020279

Garavan, H., Bartsch, H., Conway, K., Decastro, A., Goldstein, R. Z., Heeringa, S., Jernigan, T., Potter, A., Thompson, W., & Zahs, D. (2018). Recruiting the ABCD sample: Design considerations and procedures. *Developmental Cognitive Neuroscience*, *32*, 16–22. https://doi.org/10.1016/j.dcn.2018.04.004

Gatavinš, M. M., Tran, K. T., Visoki, E., Moore, T. M., Hoffman, K. W., Shilton, T., Schultz, L. M., Almasy, L., Mancini, A. D., & Barzilay, R. (2025). Adolescent depressive symptom trajectories from before to after the COVID-19 pandemic. *JAMA Network Open*, *8*(12), e2545987. https://doi.org/10.1001/jamanetworkopen.2025.45987

Hagler, D. J., Jr, Hatton, S., Cornejo, M. D., Makowski, C., Fair, D. A., Dick, A. S., Sutherland, M. T., Casey, B. J., Barch, D. M., Harms, M. P., Watts, R., Bjork, J. M., Garavan, H. P., Hilmer, L., Pung, C. J., Sicat, C. S., Kuperman, J., Bartsch, H., Xue, F., … Dale, A. M. (2019). Image processing and analysis methods for the Adolescent Brain Cognitive Development Study. *NeuroImage*, *202*, 116091. https://doi.org/10.1016/j.neuroimage.2019.116091

Kopal, J., Kumar, K., Saltoun, K., Modenato, C., Moreau, C. A., Martin-Brevet, S., Huguet, G., Jean-Louis, M., Martin, C.-O., Saci, Z., Younis, N., Tamer, P., Douard, E., Maillard, A. M., Rodriguez-Herreros, B., Pain, A., Richetin, S., Kushan, L., Silva, A. I., … Bzdok, D. (2023). Rare CNVs and phenome-wide profiling highlight brain structural divergence and phenotypical convergence. *Nature Human Behaviour*, *7*(6), 1001–1017. https://doi.org/10.1038/s41562-023-01541-9

Zhou, L., Saltoun, K., Carrier, J., Storch, K.-F., Dunbar, R. I. M., & Bzdok, D. (2025). Multimodal population study reveals the neurobiological underpinnings of chronotype. *Nature Human Behaviour*. https://doi.org/10.1038/s41562-025-02182-w
